# Residential proximity to petrol stations and risk of childhood leukemia

**DOI:** 10.1007/s10654-023-01009-0

**Published:** 2023-05-30

**Authors:** Marcella Malavolti, Carlotta Malagoli, Tommaso Filippini, Lauren A Wise, Alessio Bellelli, Giovanni Palazzi, Monica Cellini, Sofia Costanzini, Sergio Teggi, Marco Vinceti

**Affiliations:** 1 CREAGEN - Environmental, Genetic and Nutritional Epidemiology Research Center, Section of Public Health, Department of Biomedical, Metabolic and Neural Sciences, University of Modena and Reggio Emilia, Modena, Italy; 2 School of Public Health, University of California Berkeley, Berkeley, CA USA; 3 Department of Epidemiology, Boston University School of Public Health, Boston, MA USA; 4 Pediatric Oncology and Hematology Unit, Department of Medical and Surgical Sciences for Mothers, Children and Adults, University of Modena and Reggio Emilia, Modena, Italy; 5 Department of Engineering “Enzo Ferrari”, University of Modena and Reggio Emilia, Modena, Italy; 6 Department of Biomedical, Metabolic and Neural Sciences, University of Modena and Reggio Emilia, Via Giuseppe Campi 287, 41125 Modena, Italy

**Keywords:** Childhood leukemia, Benzene, Petrol station, Case-control study, Refueling activity

## Abstract

Petrol stations emit benzene and other contaminants that have been associated with an increased risk of childhood leukemia. We carried out a population-based case-control study in two provinces in Northern Italy. We enrolled 182 cases of childhood leukemia diagnosed during 1998–2019 and 726 age- and sex-matched population controls. We geocoded the addresses of child residences and 790 petrol stations located in the study area. We estimated leukemia risk according to distance from petrol stations within a 1000 m buffer and amount of supplied fuel within a buffer of 250 m from the child’s residence. We used conditional logistic regression models to approximate risk ratios (RRs) and 95% confidence intervals (CIs) for associations of interest, adjusted for potential confounders. We also modeled non-linear associations using restricted cubic splines. In secondary analyses, we restricted to acute lymphoblastic leukemia (ALL) cases and stratifed by age (<5 and ≥5 years). Compared with children who lived≥1000 m from a petrol station, the RR was 2.2 (95% CI 0.5–9.4) for children living<50 m from nearest petrol station. Associations were stronger for the ALL subtype (RR=2.9, 95% CI 0.6–13.4) and among older children (age≥5 years: RR=4.4, 95% CI 0.6–34.1; age<5 years: RR=1.6, 95% CI 0.1–19.4). Risk of leukemia was also greater (RR=1.6, 95% CI 0.7–3.3) among the most exposed participants when assigning exposure categories based on petrol stations located within 250 m of the child’s residence and total amount of gasoline delivered by the stations. Overall, residence within close proximity to a petrol station, especially one with more intense refueling activity, was associated with an increased risk of childhood leukemia, though associations were imprecise.

**Supplementary Information:**

The online version contains supplementary material available at 10.1007/s10654-023-01009-0.

## Introduction

Leukemia is the most common cancer among children. Childhood leukemia accounts for approximately 27% of all childhood cancers in the United States and 30-35% in Europe and Asia (e.g., Ireland, France, Germany, China) [1]. Acute lymphoblastic leukemia (ALL) is the most commonly diagnosed childhood cancer worldwide [2] and is more frequent in European and Hispanic populations [3]. According to data from populations covered by high-quality cancer registries, the incidence of ALL has been estimated at 46.4 per 1 million children (i.e., one third of all new diagnoses) [3].

Though epidemiological studies have identified several potential genetic and environmental risk factors for childhood leukemia, uncertainties still surround their causes, the presence of dose-response relations, and the presence of thresholds for environmental contaminants to increase disease risk [4–16]. Air pollution generated by motorized traffic and industrial sources is one of these putative risk factors [17]. The International Agency for Research on Cancer (IARC) classified outdoor air pollution and particulate matter from outdoor air pollution as carcinogenic to humans (IARC Group 1), based on sufficient evidence of carcinogenicity in humans and experimental animals and strong mechanistic evidence. Among traffic pollutants, there is benzene, a designated Group I carcinogen by the IARC that is associated with adult leukemia and lymphomas [18–20], and there is consistent evidence indicating that benzene exposure increases the risk of childhood leukemia [21]. A harmful effect of benzene is still conceivable even at the much lower air pollution levels documented in western countries during the last decades [22].

In urban areas, the highest concentrations of atmospheric pollutants have been found near high-traffic roads. However, since pollutants are emitted during fuel refueling activities and petrol leaks, petrol stations are also considered potential sources of exposure to air pollutants, such as 1,3-butadiene and in particular benzene [23, 24]. Primary petrol compounds enter the air of gas stations due to high evaporation of gasoline. A recent study evaluated the concentration of these compounds in the ambient air of gas stations [23] and found benzene concentrations ranged from about 1 to more than 5 ppm, higher than the recommended exposure limit, 0.1 ppm time weighted average, by The National Institute for Occupational Safety and Health [25, 26].

Some studies have assessed the contribution of petrol refueling stations to the pollutants concentrations in their wider vicinity, finding higher levels of pollutants not only close to the pumps, but also throughout the whole service station area and some distance beyond (50–100 m) [24, 27–30], depending on station fueling activity and meteorological conditions [29, 31]. To our knowledge, however, no studies have considered the station’s activity when estimating effects of exposure on disease risk, despite the levels of pollutant concentrations around stations being strongly dependent on the amount of fuel delivered [31, 32].

We assessed the extent to which residential exposure to gasoline service stations was associated with risk of childhood leukemia in Northern Italy, by considering both distance from the stations located within proximity of the residence and gas station activity. We also critically summarized existing epidemiologic evidence on the risk of childhood leukemia following long-term exposure to gasoline service stations, updating a previous meta-analysis on this topic [33].

## Methods

### Case and control selection

Following the Ethics Committee approval [14, 16, 34], we have identified all newly-diagnosed cases of childhood leukemia (ICD-9 codes 204–208) in the 0–14 aged population of Modena and Reggio Emilia, two provinces in Northern Italy (population around 1,200,000), from 1998 to 2019. Details for case and control identification have been described elsewhere in detail [35]. Briefly, we identified the cases through the Italian hospital-based registry of childhood malignancies, available from the Italian Association of Pediatric Emato-Oncology (AIEOP) and capturing all cancer cases arising in Italy [36]. The referent population included four children for each case, matched on sex, year of birth, and province of residence during the year of diagnosis, randomly selected among those enrolled in the National Health Service directory of the Modena and Reggio Emilia provinces, where all residents are compulsorily registered.

We collected data on residential address at time of diagnosis for cases from the AIEOP databased, and in the corresponding year for their matched controls using the historical population database of the National Health Service. We geocoded the home buildings within a Geographical Information System (GIS) using Arc-GIS software (version 9.2, ESRI, Redlands, CA 2006). The satellite coordinates of the residences were retrieved using methodology based on an official geocoding database made available by the Modena and Reggio Emilia Province and, for addresses not included in the database, through the Google Earth App or direct *in loco* measurement using a portable GPS device (GPSmap 60CSx, Garmin Int. Corp., Olathe, KS) [16, 34, 35]. To identify petrol stations, we obtained information from the Trade Observatory of the Emilia-Romagna Region about all the 859 facilities located in the territory of Modena and Reggio Emilia provinces. All stations were georeferenced in the GIS through Google Earth or *in loco* measurements, focusing on the exact position of the pumps whenever possible, or to the geometric center of the station area.

### Exposure to petrol station

We assessed exposure to petrol stations in two ways. First, petrol station exposure was expressed as distance from home to the nearest petrol station divided into categories with the following cut points: 50, 200, 500, 1000 and over 1000 m.

Secondly, we estimated the activity of each station in terms of fuel sold over an entire year, and we assessed child exposure to all stations located up to 1000 m around the residences. For this purpose, through record-linkage with the database provided by the Trade Observatory of the Emilia-Romagna Region, we retrieved the average daily quantity (L/day) of fuel sold in each petrol station in the year of leukemia diagnosis for cases and their matched controls. The total average daily quantity of fuel supplied by all the petrol stations located within 250 m buffer around residence was computed for each study participant. For this analysis, we then categorized petrol station exposure based on the amount of supplied fuel as follows: 0 = no petrol stations within the 250 m-buffer; 1 = fuel supply until 149 L/day within the 250 m-buffer; 2 = fuel supply ≥ 150 until 999 L/day within the 250 m-buffer; and 3 = fuel supply ≥ 1000 L/day within the 250 m-buffer.

### Confounders

We considered several potential confounders in multivariable analysis. We modeled outdoor air concentration of PM_10_ at the residence of each child, using the CAlifornia LINE Source Dispersion Model, version 4 (CALINE4 - Sacramento CA, Dept. of transportation, Division of New Technology and Research, 1989), a line source air quality model, based on vehicular traffic flow parameters and meteorological data [34]. We also modeled the magnetic fields generated by the 132 + kV power lines possibly located close to the child’s residence in the study territory using a methodology previously validated and explained in detail [16, 37]. We also included as adjustment factor to the multivariable analysis the presence of indoor transformer stations in the building of residence, using information about high-voltage power line net and transformer stations in the two study provinces made available by the Emilia-Romagna Regional Agency for Environmental Prevention and Energy (ARPAE). We then determined the urban and arable crop by calculating the percentage of the land use type in proximity to each geocoded home, based on a circular area with a radius of 100 m around the residence of each child according to the Land Use Map 2014 for both Modena and Reggio Emilia provinces [14, 38, 39].

We also collected information on socio-demographic variables maternal age and ethnicity using birth certificate data provided by the Local Health Authorities of Modena and Reggio Emilia, and parental annual income for the index year by the Italian Revenue Agency of the Ministry of Finance.

### Data analysis

We estimated the risk ratio (RR) of childhood leukemia in relation to categorical distance (categorized as < 20, 50 - <200, 200 - <500, 500 - <1000 and ≥ 1000 m) to the nearest petrol station by computing the disease odds ratio and its 95% confidence interval (CI) using conditional logistic regression models, with matching for age, sex and province of residence. In the multivariable models, we included as potential confounders the following variables: modeled PM_10_ concentrations, calculated ELF-MF (categorized as < 0.1, 0.1 - <0.2, 0.2 - <0.4 and ≥ 0.4 µT) [16], presence of electric transformer rooms near the building of residence (categorized as < 5 m, ≥ 5 - <10 m, ≥ 10 - <20 m, - ≥20 m), percentage of urban area providing information related to the type of neighborhood within the 100 m-buffer around the residence [35], and percentage of arable crops within the 100 m-buffer around the residence (continuous) as related to pesticide exposure [14]. In addition, in a subgroup analysis for participants having additional information available, we added maternal ethnicity (categorized as white, Black or Asian), father income, and maternal age at delivery (continuous). We also conducted subgroup analyses by child’s age of diagnosis (< 5 and ≥ 5 years), and restricted to ALL, the only cancer subtype with sufficient numbers for meaningful analyses. We used restricted cubic splines to model the shape of the association. We selected the number of knots using the Akaike Information Criterion (AIC) and the knot-placement method [40] to assess the association between residential distance from the nearest petrol station and RR of leukemia through a nonlinear model based on restricted cubic splines, using three knots at fixed distances (50, 200, and 500 m) and ≥ 1000 m as the reference.

### Updated systematic review and meta-analysis

We performed a systematic literature search (PROSPERO registration no. CRD42023402919) using online databases PubMed/MEDLINE, Web of Science and EMBASE from inception up to April 3, 2023 according the PRISMA guidelines [41]. We also used citation chasing methods namely backward and forward reference list scanning to retrieve additional eligible papers. Two authors (TF and MV) performed the screening of title/abstract and then of the full-text with the help of a third author (MM) to solve disagreement. According PECOS (population, exposure, comparison, outcome and study design) statement, we searched all observational studies that have investigated the risk of childhood leukemia in relation to exposure to petrol station using either proximity of children residence or modelled exposure. We used keywords related to ‘petrol’ or ‘gasoline station’ and ‘childhood’ or ‘infant leukemia’. Details of literature search are reported in Supplemental Table S1. We assessed the risk of bias (RoB) of included studies using the Newcastle - Ottawa quality assessment scale (NOS). Details of criteria for study evaluation are reported in Supplemental Table S2. Two authors independently performed the RoB assessment (MM and TF), with discrepancies solved based on the review of a third author (MV). We carried out a highest versus lowest exposure meta-analysis of all eligible studies using a random-effects model, and we performed stratified analysis according exposure assessment method (i.e., using questionnaires or georeferencing data). Finally, we assessed potential for publication bias using a funnel plot and Egger’s test.

## Results

The study enrolled 183 incident cases of childhood leukemia, of which 148 were cases of lymphoblastic leukemia ALL, and 732 matched controls. We excluded one case (alongside the respective controls) and two additional eligible controls due to missing residential information. The final analysis included 182 cases (98 males and 84 females) and 726 age-and sex-matched population controls. The average age at diagnosis was 6.2 years (standard deviation: 3.9), with corresponding median value of 5.7 years (interquartile range-IQR: 3.0–9.0). The median values (IQR) for cases and controls of the adjustment variables were: fuel supply (L/day) within the 1000 m-buffer = 804 (0-2454) and 868 (0-2255); PM_10_ (µg/m^3^) = 4.9 (2.4–8.3) and 4.6 (2.0-7.8); urban area within the 100 m-buffer around the residence = 0.6 (0.4–0.8) and 0.6 (0.4–0.8); arable crops within the 100 m-buffer around the residence = 0.0 (0.0-0.1) and 0.0 (0.0-0.1), respectively. Distribution for ELF-MF was categorized as follows: <0.1 µT for 180 cases and 725 controls; 0.1 - <0.2 µT for 0 cases and 1 control; 0.2 - <0.4 µT for 1 case and 0 controls and ≥ 0.4 µT 1 case and 0 controls. Electric transformer rooms near the building of residence were categorized for cases and controls as follows: ≥20 m for 178 cases and 709 controls; ≥10 and < 20 m for 2 cases and 10 controls; ≥5 and < 10 m for 1 case and 4 controls; <5 m for 1 case and 3 controls. Data on the distribution of cases and controls by residential proximity to the nearest petrol station and by exposure category are reported in Table 1.

**Table 1:** Distribution of study population (cases and controls) by distance from residence to the nearest petrol station and category of exposure to air pollutants emissions from the nearby station

	All leukemias			Acute lymphoblastic leukemia (ALL)		
	All subjects	< 5 years	≥ 5 years	All subjects	< 5 years	≥ 5 years
Distance to the nearest petrol station, meters						
≥ 1000 (Referent)	48/185	23/83	25/102	38/145	19/63	19/82
500–< 1000	53/214	22/93	31/121	44/177	17/72	27/105
200–< 500	58/242	25/113	33/129	47/202	20/94	27/108
50–< 200	20/80	12/40	8/40	16/62	9/33	7/29
< 50	3/5	1/3	2/2	3/4	1/2	2/2
All subjects	182/726	83/332	99/394	148/590	66/264	82/326
Class of exposure^a^						
0 (Referent)	146/608	64/278	82/330	118/497	51/219	67/278
1	4/12	2/6	2/6	4/11	2/5	2/6
2	21/76	14/34	7/42	18/59	11/28	7/31
3	11/30	3/14	8/16	8/23	2/12	6/11
All subjects	182/726	83/332	99/394	148/590	66/264	82/326

^a^Exposure category: 0 = no petrol stations within 250 m-buffer; 1 = fuel supply until 149 L/day within 250 m-buffer; 2 = fuel supply ≥ 150 until 999 L/day within 250 m-buffer; 3 = fuel supply ≥ 1000 L/day within 250 m-buffer

RRs for leukemia risk according to residential proximity to the nearest petrol station are shown in Table 2. Compared to those living ≥ 1000 m, RRs for children living < 50 m were 2.3 (95% CI 0.5–10.0) and 2.2 (95% CI 0.5–9.4) in crude and adjusted analyses, respectively. Corresponding RRs were stronger in analyses confined to ALL cases: 2.8 (95% CI 0.6–13.2) and 2.9 (95% CI 0.6–13.4), respectively.

**Table 2:** Distance from residence to the nearest petrol station and risk of childhood leukemia

Distance to the nearest petrol station, m	All subjects	Age < 5 years	Age ≥ 5 years
	RR (95% CI)	RR (95% CI)	RR (95% CI)
*Bivariate model*^a^			
All leukemias			
≥ 1000 (Referent)	1.0	1.0	1.0
500–< 1000	1.0 (0.6–1.6)	0.9 (0.4–1.6)	1.1 (0.6–2.0)
200–< 500	0.9 (0.6–1.4)	0.8 (0.4–1.6)	1.1 (0.6–2.0)
50–< 200	1.0 (0.5–1.8)	1.1 (0.5–2.4)	0.9 (0.4–2.1)
< 50	2.3 (0.5–10.0)	1.2 (0.1–12.2)	4.3 (0.6–32.4)
ALL			
≥ 1000 (Referent)	1.0	1.0	1.0
500–< 1000	1.0 (0.6–1.6)	0.7 (0.3–1.6)	1.2 (0.6–2.3)
200–< 500	0.9 (0.6–1.5)	0.7 (0.4–1.5)	1.2 (0.6–2.3)
50–< 200	1.0 (0.5–2.0)	0.9 (0.4–2.3)	1.2 (0.4–3.1)
< 50	2.8 (0.6–13.2)	1.6 (0.1–18.8)	4.7 (0.6–36.0)
*Multivariable model*^b^			
All leukemias			
≥ 1000 (Referent)	1.0	1.0	1.0
500–< 1000	0.9 (0.6–1.5)	0.8 (0.4–1.6)	1.1 (0.6–2.1)
200–< 500	0.9 (0.5–1.5)	0.8 (0.4–1.7)	1.1 (0.5–2.2)
50–< 200	1.0 (0.5–1.9)	1.0 (0.4–2.5)	1.0 (0.4–2.7)
< 50	2.2 (0.5–9.4)	1.1 (0.1–11.4)	3.6 (0.5–27.6)
ALL			
≥ 1000 (Referent)	1.0	1.0	1.0
500–< 1000	1.0 (0.6–1.7)	0.7 (0.3–1.6)	1.3 (0.6–2.7)
200–< 500	1.0 (0.6–1.7)	0.7 (0.3–1.7)	1.4 (0.6–2.9)
50–< 200	1.2 (0.5–2.5)	0.9 (0.3–2.5)	1.7 (0.6–5.0)
< 50	2.9 (0.6–13.4)	1.6 (0.1–19.4)	4.4 (0.6–34.1)

*ALL* acute lymphoblastic leukemia

^a^Matched on sex, age and province of residence

^b^Adjusted for fuel supply within the 1000 m-buffer, PM_10_, ELF-MF from high-voltage power lines, indoor transformer stations, urban area and arable crops

The age-stratified results are presented in Table 2. Focusing on children whose residence was extremely close to petrol station (< 50 m), we found an increased risk among older children (age ≥ 5 years) of 4.3 (95% CI 0.6–32.4) compared with 1.2 (95% CI 0.1–12.2) among younger children (age < 5 years) in the bivariate model. When limiting the analysis to ALL cases in the bivariate model, RRs were 1.6 (95% CI 0.1–18.8) among younger children and 4.7 (95% CI 0.6–36.0) among older children. In multivariable analysis, we found relatively similar RRs for all leukemia cases and as well as ALL cases (Table 2).

**Fig. 1:**
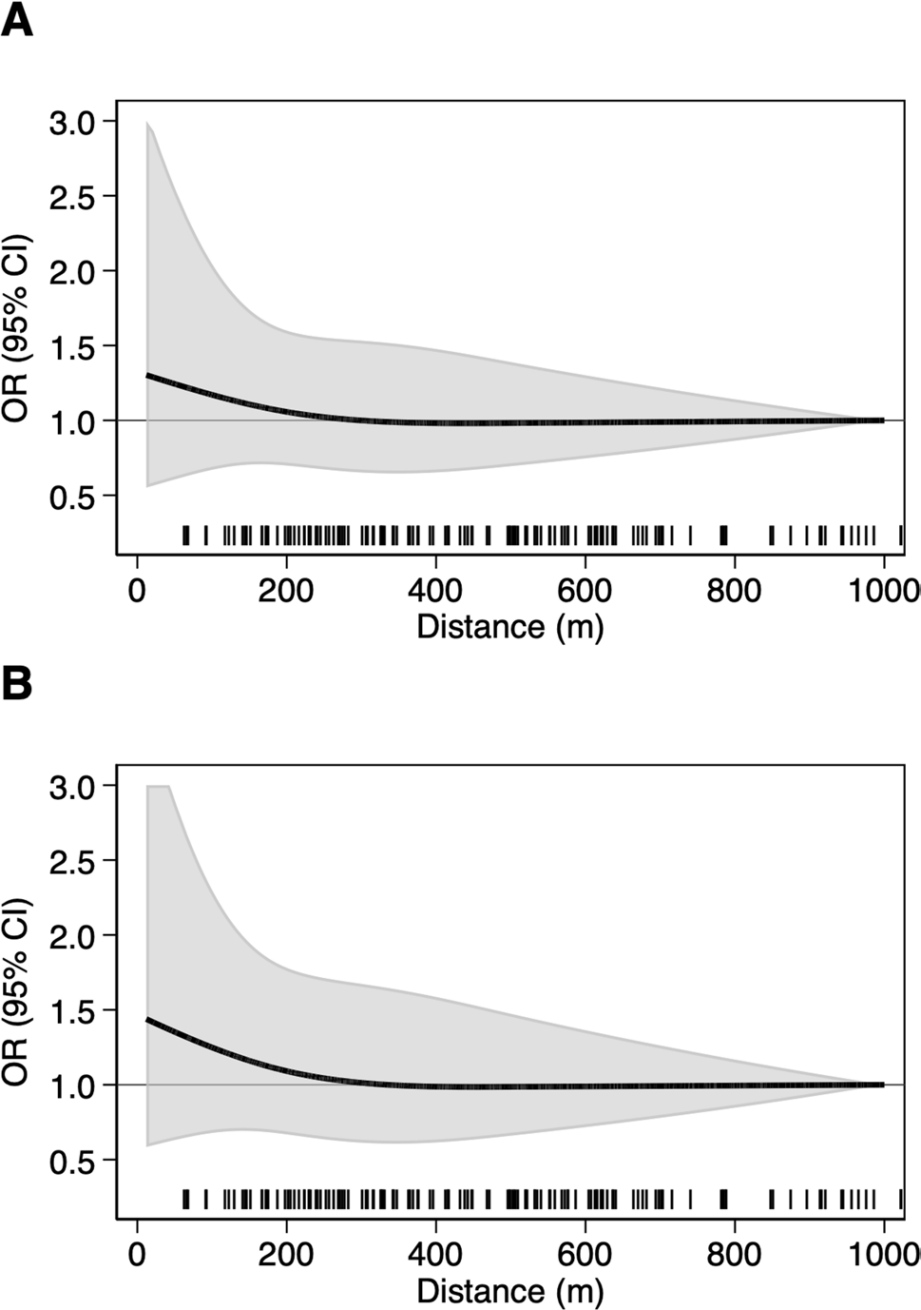
Spline regression analysis assessing the risk ratio of childhood leukemia according to distance of child’s residence from the closest petrol station. Restricted cubic spline with three knots at fixed distances (50, 200, and 500 m). **A** Bivariate model; **B** Multivariable model

**Fig. 2:**
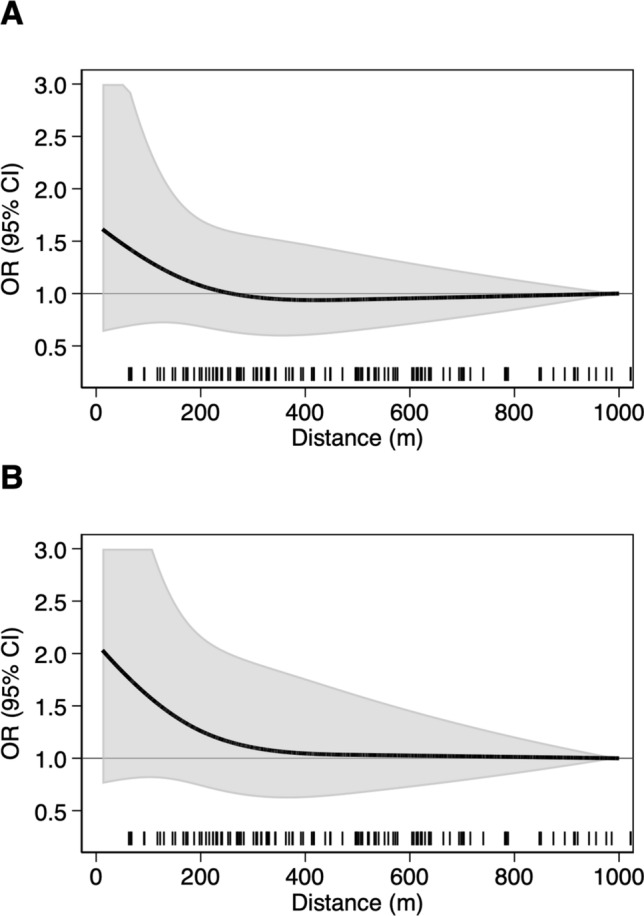
Spline regression analysis assessing the risk ratio of childhood acute lymphoblastic leukemia (ALL) according to distance of child’s residence from petrol station. Restricted cubic spline with three knots at fixed distances (50, 200, and 500 m). **A** Bivariate model; **B** Multivariable model

In spline regression analyses for overall leukemia (Fig. 1) and ALL (Fig. 2), residential proximity to a petrol station was positively associated with leukemia risk only within close proximity of the station (< 50 m): RR = 1.4 (95% CI 0.6–2.9).

When we considered all the petrol stations located within 250 m of the child’s residence, as well as the total amount of gasoline sold by the station the year before the index year, RRs for leukemia were 1.6 (95% CI 0.8–3.2) and 1.6 (95% CI 0.7–3.3) in bivariate and multivariable analyses, respectively, for the highest category of exposure (Table 3), with no indication of monotonic relation across the exposure categories. Corresponding RR estimates for ALL were 1.5 (0.6–3.5) and 1.5 (0.6–3.8). Older children (age ≥ 5 years) living inside buffer of 250 m with petrol stations that have sold more than 1000 L/day had a higher risk of leukemia overall (bivariate and multivariable models: RR = 2.1, 95% CI 0.8–5.1 and RR = 2.4, 95% CI 0.9–6.1, respectively), and of ALL (RR = 2.4, 95% CI 0.8–7.2 in the bivariate model and RR = 3.4, 95% CI 1.0-11.1 in the multivariable model). In the remaining categories of exposure, there was no clear association, neither evidence of dose-response trends. Sensitivity analyses among subjects with complete data on demographic variables, specifically maternal ethnicity and paternal income, showed similar though less precise results (Supplemental Table S3) when compared with the overall analysis and the analysis among the same subgroup without adjusting for these additional variables.

**Table 3:** Class of exposure within a 250-meter buffer from petrol stations considering gasoline distributed, and risk of childhood leukemia

Class of exposure	All subjects	< 5 years	≥ 5 years
	RR (95% CI)	RR (95% CI)	RR (95% CI)
*Bivariate model*^a^			
All leukemias			
0 (Referent)	1.0	1.0	1.0
1	1.4 (0.4–4.3)	1.6 (0.3–8.4)	1.2 (0.2–6.2)
2	1.2 (0.7–1.9)	1.8 (0.9–3.6)	0.7 (0.3–1.6)
3	1.6 (0.8–3.2)	0.9 (0.2–3.2)	2.1 (0.8–5.1)
ALL			
0 (Referent)	1.0	1.0	1.0
1	1.5 (0.5–4.8)	2.0 (0.4–11.0)	1.3 (0.3–6.4)
2	1.3 (0.7–2.3)	1.7 (0.8–3.8)	1.0 (0.4–2.4)
3	1.5 (0.6–3.5)	0.6 (0.1–3.0)	2.4 (0.8–7.2)
*Multivariable model*^b^			
All leukemias			
0 (Referent)	1.0	1.0	1.0
1	1.4 (0.4–4.4)	1.5 (0.3–7.9)	1.4 (0.3–7.5)
2	1.2 (0.7–2.1)	2.0 (1.0–4.1)	0.7 (0.3–1.7)
3	1.6 (0.7–3.3)	0.8 (0.2–3.1)	2.4 (0.9–6.1)
ALL			
0 (Referent)	1.0	1.0	1.0
1	1.6 (0.5–5.0)	1.8 (0.3–10.1)	1.5 (0.3–8.1)
2	1.4 (0.8–2.5)	2.0 (0.9–4.5)	1.0 (0.4–2.6)
3	1.5 (0.6–3.8)	0.6 (0.1–3.0)	3.4 (1.0–11.1)

*ALL* acute lymphoblastic leukemia

^a^Matched on sex, age and province of residence

^b^Adjusted for PM_10_, ELF-MF from high-voltage power lines, indoor transformer stations, urban area and arable crops

^c^Class of exposure: 0 = no petrol stations within the 250 m-buffer; 1 = fuel supply until 149 L/day within the 250 m-buffer; 2 = fuel supply ≥ 150 until 999 L/day within the 250 m-buffer; 3 = fuel supply ≥ 1000 L/day within the 250 m-buffer

In the systematic review and meta-analysis, our literature search retrieved 31 records after removal of duplicates. We then excluded 20 studies after title/abstract screening and an additional six studies after full-text evaluation as they assessed benzene exposure during parental occupation only (n = 1), petrol station exposure was not evaluated (n = 2), or were conference abstract (n = 1) or commentaries (n = 2) (Supplemental Figure S2). We retrieved one additional study [42] through citation chasing, leading to a total of six studies for analysis not including the present study. Characteristics of retrieved studies are reported in Table 4. The ages of the study populations for all in the range 0–14 years, with dates of diagnosis ranging from 1985 to 2019. All studies had a case-control design, including a case-cohort study [33]. Three studies investigated exposure to petrol station using questionnaire [42–44], although one study assessed exposure to both petrol station and car repair garage [43]. One study validated questionnaire-based exposure using georeferencing data [44]. The three remaining studies used georeferencing data for exposure assessment [33, 45, 46], one in particular through evaluation of petrol station density (number of stations per km^2^). Results of the bias assessment are reported in Supplemental Table S4.

**Table 4:** Characteristics of studies included in the systematic review

Reference	Design	Region	Cases/non cases	Age (years)	Diagnosis	Assessment	Risk estimate	Adjusting factors
Abdul Rahman 2008 [42]	Case-control	Klang Valley, Malaysia	128/128	< 15	2001–2007 acute leukemia	Questionnaire: distance of residence at the time of diagnosis from a petrol station ≤ 1 km vs. >1 km	OR: 0.84 (95% CI 0.50–1.41)	Crude
Brosselin 2009 [44]	Case-control	France	765/1681	< 15	2003–2004 acute leukemia	Questionnaire: Ever (vs. never) lived in proximity (not described in detail) to a petrol station and/or automotive repair garage Data validated using georeferencing data	Repair garage: OR: 1.4 (95% CI 1.0–2.0) Petrol station: OR: 1.9 (95% CI 1.2–3.0) Any: OR: 1.6 (95% CI: 1.2–2.2) Any by period: Childhood: OR: 1.3 (95% CI 0.9–1.9) During pregnancy: OR 1.4 (95% CI 1.0–2.1) Both: OR: 1.7 (95% CI: 0.9–3.4)	Age, sex, number of children < 15 years living in the household, and stratification variables
					ALL		Repair garage: OR: 1.5 (95% CI: 0.9–2.3) Petrol station: OR: 2.0 (95% CI: 1.0–4.0) Any: OR: 1.6 (95% CI 1.9–2.3) Both: OR: 1.8 (95% CI 0.9–3.5)	
					AML		Repair garage: OR: 0.8 (95% CI: 0.2–2.5) Petrol station: OR: 2.5 (95% CI: 0.7–8.8) Any: OR: 1.1 (95% CI 0.5–2.5) Both: OR: 0.8 (95% CI 0.1–6.2)	
Harrison 1999 [46]	Case-control	West Midlands, UK	130/251	0–15	1990–1994 acute leukemia	Georeferencing data: petrol station proximity (≤ 100 m vs. >100 m)	OR: 1.99 (95% CI 0.73–5.43) IR: 1.48 (95% CI 0.65–2.93)	Crude
Mazzei 2022 [33]	Case-cohort	Swiss	1880/18,800	0–15	1985–2015	Georeferencing data: petrol station distance < 50 m vs. ≥500 m	OR: 1.08 (95% CI 0.46–2.51)	Age- and sex-matched. Adjusted for NO_2_, distance to the nearest highway, socio-economic position of the immediate neighborhood area, degree of urbanization of the municipality of residence, and years of existence of a general cantonal cancer registry
Steffen 2004 [43]	Case-control	Nancy, Lille, Lyon and Paris, France	280/285	0–14	1995–1999 acute leukemia	Face-to-face interview: vicinity (< 50 m for traffic) of dwellings neighboring including petrol station or repair garage	Childhood: OR: 4.0 (95% CI 1.5–10.3) During pregnancy: OR: 2.2 (95% CI 0.9–5.7)	Age, sex, center, and ethnic origin
					ALL		Childhood: OR: 7.7 (95% CI 1.7–34.3)	
					AML		Childhood: OR: 3.6 (95% CI 1.3–9.9)	
Weng 2009 [45]	Case-control	Taiwan	729/729	0–14	1996–2006 acute leukemia	Petrol station density (n/km^2^) in tertiles: T1: ≤0.149 (median 0.065); T2: 0.150–0.395 (0.225); T3: 0.399–2.692 (0.585)	T2 - OR: 1.45 (95% CI 1.06–1.98) T3 - OR: 1.91 (95% CI 1.29–2.82)	Sex, year of birth, year of death, and urbanization level
This study	Case-control	Italy	182/726	0–14	1998–2019 acute leukemia	Georeferencing data: petrol station proximity (≤ 50 m vs. >1000 m) Modeling using also fuel supply data divided in four categories	Proximity 2.2 (95% CI 0.5–9.4) Modeling 1.6 (95% CI 0.7–3.3)	Age- and sex-matched. Adjusted for PM_10_, ELF-MF from high-voltage power lines, indoor transformer stations, urban area and arable crop
			148/590		ALL		Proximity 2.9 (95% CI 0.6–13.4) Modeling 1.5 (95% CI 0.6–3.8)	

**Fig. 3:**
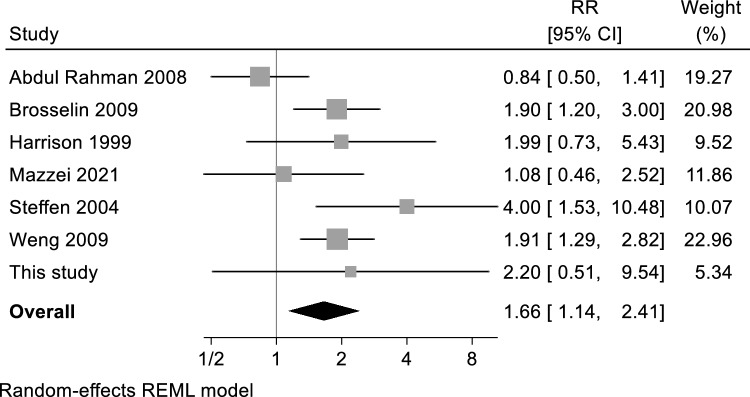
Forest-plot of the meta-analysis of the association between petrol station exposure and childhood leukemia risk. The area of each grey square is proportional to the inverse of the variance of the estimated log risk ratio (RR) and horizontal lines represent their 95% confidence intervals (Cis). The black diamond represents the combined RR using the random-effects restricted maximum likelihood (REML) model. The solid vertical line represents RR = 1

Meta-analysis of the results of the aforementioned six studies plus those generated by the present study are reported in Fig. 3, showing a summary RR of 1.66 (95% CI 1.14–2.41). Analyses stratified by modality of exposure assessment yielded similar results, with lower precision for studies based on questionnaire data (Supplemental Figure S3). Sensitivity analysis excluding the study assessing also proximity of car repair garages showed consistent results, yielding a summary RR of 1.50 (95% CI 1.05–2.15) (Supplemental Figure S4). The sensitivity analysis restricted to four “high-quality” studies (NOS score ≥ 8) still showed an elevated disease risk with RR = 1.80 (95% CI 1.37–2.38) (Supplemental Figure S5). The funnel plot showed some evidence of publication bias (Supplemental Figure S6).

## Discussion

In this study, we examined childhood leukemia risk in relation to proximity to petrol stations. We observed an increased leukemia risk, though imprecise, when assessing exposure as distance from residence (< 50 m) to the nearest petrol station, while the excess risk associated with the intensity of activity of all stations located < 250 m from the residence was not as large. In both analyses no dose-response relation emerged, since a clear excess risk occurred only in the highest exposure category, suggesting the occurrence of a threshold of exposure to the fugitive chemical emissions from fuel pumps, heightening disease risk, mainly based on the distance from the station. This suggests a far higher relevance of close residential proximity to the gas station when compared with ‘moderate’ proximity and with the overall station refueling activity, in terms of increasing childhood leukemia risk.

While there are no previous studies based on the station activity, to the best of our knowledge, our results on residential distance from petrol station are consistent with three of the four studies carried out on the same topic. In a UK study [46] that estimated childhood cancer risks in relation to proximity to main roads and petrol stations, a slight increase in leukemia risk was found within 100 m from a petrol station (OR = 1.5, 95% CI 0.6–2.9). In a hospital-based case-control study in France [43], residence close to a petrol station or a repair garage during childhood was strongly associated with excess risk of childhood leukemia (OR = 4.0, 95% CI 1.5 to 10.3). This association was even stronger for acute non-lymphocytic leukemia (OR = 7.7, 95% CI 1.7 to 34.3), and was not altered by adjustment for potential confounding factors. In 2009, Brosselin et al. [44] reported results of a large national registry-based case-control study ESCALE in France (2003–2004) indicating a strong positive association between living in a residence adjoining of a garage or petrol station and acute childhood leukemia. However, these studies differed from ours regarding exposure definitions and assessment: while we classified children living within 50 m of a petrol station in the highest exposure category, the English study used a 100 m threshold and the French studies considered only children living close to a gas station, without specifying the exact distance, also assessing exposure through interviews with the children’s mothers and thus potentially affected by recall bias [43, 44]. In a case-control study carried out in Malaysia [42], and based on data collected through questionnaires, no association emerged, though the cut point used to refine residential exposure was quite large, not comparable to that used in the other studies including our one and likely inadequate to detect any association (≤ 1 km vs. >1 km from a petrol station).

An additional case-control study [45] revealed an exposure-response relation between petrol station density (per square kilometer), as a marker of traffic-related air pollution, and the risk of leukemia in young children. A nationwide case-control study carried out in Switzerland (during 1985–2015) found evidence of an increased risk of childhood cancer (all diagnoses combined) among children living in close vicinity (< 50 m vs. ≥500 m) of petrol stations [33].

A relevant distinct feature of the present analysis is the integration of data on petrol station activity along with its distance from the child’s residence. There is clear evidence from studies of gas station workers that their exposure to air pollutants is positively related to the volume of refueling in petrol stations, as well as the confinement of pollutants in semi-closed spaces of the work place [25, 47–49].

Our results showed a direct relation between residing in close proximity to a petrol station and risk of leukemia, both based on fixed cut points of distance from the stations and modeling exposure through a combination of distance and gas station activity. Associations were strongest for ALL cases in older children (age ≥ 5 years), with a two-fold increased risk for children in the category of major exposure (< 50 m) and a four-fold increased risk among children diagnosed after 5 years. The latter finding might be ascribed to a higher cumulative exposure among older children, due to both their age and to their tendency to spend more time outside. Such excess risk may persist up to 250 m from the gas station also depending on their activity, a plausible finding given the results of air monitoring studies [31, 50].

In this study, we assessed exposure without requiring any active participation by study participants and their families, nor by petrol station personnel, thus avoiding selection and information bias, as individual participation was not needed. We also carried out the exposure assessment in a blinded manner with reference to the case and control status of the participants. A potential limitation of our study is that information on the activity of each petrol station was available during 1998–2017 only. Since our study includes children with diagnoses that occurred from 1998 to 2019, we decided to consider, for the last two years 2018-19 and for each station, the fuel supply corresponding to the most recent year available: 2017. Residual or unmeasured confounding could have also been possible, and some demographic characteristics were unavailable for many study participants as well as complete information about medical imaging procedures [4]. A sensitivity analysis limited to study participants accounting for all potential confounders measured yielded similar results to the analysis carried out without such more comprehensive adjustment, both in this subgroup and in the entire study population, suggesting that the demographic factors for which we lacked complete information were not a major source of confounding. However, we may not have collected and controlled for all relevant confounders of the associations (e.g., using paternal income as a proxy for household income, or lacking information about exposure to ionizing radiation for diagnostic purposes), and therefore some effect of residual confounding in biasing our results could not be entirely ruled out [51]. We also acknowledge the statistical instability of our risk estimates, due to the very limited number of exposed children, a limitation suggesting caution in interpreting our results, though being consistent with the results from the other comparable studies as reflected by the pooled estimates of the meta-analysis. Finally, we acknowledge the potential for exposure misclassification associated with lack of historical residential stability or time spent at home (e.g., a substantial part of the daytime hours may have spent at a different residence (e.g., grandparents’ home, school, or day care). However, while we could not comprehensively assess residential mobility or time spent at one’s residence since the study design did not include a direct contact with children’s families, residential stability of the study participants was likely to be high (> 70% for all children and > 82% for children less than 5 years) based on data previously ascertained in subgroup of the study population [14, 16, 34].

## Electronic supplementary material

Below is the link to the electronic supplementary material.

Supplementary Material 1

## Abbreviations

AIC: Akaike Information Criterion
AIEOP: Associazione Italiana Ematologia Oncologia Pediatrica
ARPAE: Regional Agency for Environmental Protection and Energy
CI: Confidence interval
GIS: Geographical Information System
ICD-9: International Classification of Diseases, 9th Edition
µT: MicroTesla
RR: Relative risk
