# Supplementary material for: Residential proximity to petrol stations and risk of childhood leukemia
Source: Eur J Epidemiol. 2023 May 30;38(7):771–82. doi: 10.1007/s10654-023-01009-0 (PMC10275799; doi:10.1007/s10654-023-01009-0)
Supplement: Supplementary file 1 — Supplementary Material 1 [file 10654_2023_1009_MOESM1_ESM.docx]

**SUPPLEMENTAL MATERIAL**

**Supplemental Table S1**. Database literature search in online databases.

| **Database** | **Search** |
| --- | --- |
| PubMed | (petrol station OR gasoline station OR gas station) AND ((leukemia[MH] OR leukaemia[TIAB] OR leukemia[TIAB] OR childhood leukemia[TIAB]) AND (child[MH] OR infant[MH] OR adolescent[MH] OR child[TIAB] OR infant[TIAB] OR adolescent[TIAB] OR childhood[TIAB] OR children[TIAB])) NOT ((animals [MH] OR plants [MH]) NOT humans [MH]) NOT review[PT] |
| Web of Science | ((TS=(petrol station) OR TI=(petrol station)) OR (TS=(gasoline station) OR TI=(gasoline station)) OR (TS=(gas station) OR TI=(gas station)) ) AND ((TS=(leukemia) OR TI=(leukemia) OR TS=(leukaemia) OR TI=(leukaemia)) OR TI=(childhood leukemia) ) AND ( TS=(child OR infant OR adolescent) OR TI=(child OR infant OR adolescent)) |
| EMBASE | ('petrol station' OR (('petrol'/exp OR petrol) AND station) OR 'gasoline station' OR (('gasoline'/exp OR gasoline) AND station) OR 'gas station' OR (('gas'/exp OR gas) AND station)) AND ('childhood leukemia'/exp OR 'childhood leukemia' OR (('childhood'/exp OR childhood) AND ('leukemia'/exp OR leukemia)) OR (('leukemia'/exp OR leukemia) AND ('child'/exp OR child OR 'adolescent'/exp OR adolescent))) NOT ('review'/exp OR review) |

**Supplemental Table S2.** Newcastle - Ottawa quality assessment scale for included studies: details used for study score assignment. High quality choices are identified with a ‘star’ (i.e. asterisk). A maximum of one ‘star’ for each item within the ‘Selection’ and ‘Exposure’ categories; maximum of two ‘stars’ for ‘Comparability’ can be identified.

| **(A) Newcastle - Ottawa quality assessment scale for case-control studies** | |
| --- | --- |
| **Selection**  1) Is the case definition adequate?   1. yes, with independent validation* 2. yes, e.g., record linkage or based on self-report 3. no description   2) Representativeness of the cases   1. consecutive or obviously representative series of cases* 2. potential for selection biases or not stated   3) Selection of Controls   1. community controls* 2. hospital controls 3. no description or not representative of the population   4) Definition of Controls   1. no history of disease (endpoint)* 2. no description of source | **Comparability (both case-control and cohort studies)**  1) Comparability of cases and controls/cohorts on the basis of the design or analysis: (yes/no answer)   - study controls for age (y*/n) - study controls for urbanization level (y*/n) |
|  | **Exposure (for case-control studies)**  1) Ascertainment of exposure   1. secure record (e.g. surgical records)* 2. structured interview where blind to case/control status* 3. interview not blinded to case/control status 4. written self-report or medical record only 5. no description   2) Same method of ascertainment for cases and controls   1. yes* 2. no   3) Non-response rate   1. same rate for both groups* 2. non-respondents described 3. rate different and no designation |

**Supplemental Table S3**. Restricted analysis to subjects who have all the adjustment variables (environmental and demographic).

| **Distance to nearest**  **petrol station, m** |  | **Crude analysis** | **Adjusted analysis for demographic^1^ and environmental^2^ variables** | **Adjusted analysis for only demographic variables** | **Adjusted analysis for only environmental variables** |
| --- | --- | --- | --- | --- | --- |
|  | **Cases/controls** | **RR (95% CI)** | **RR (95% CI)** | **RR (95% CI)** | **RR (95% CI)** |
| ≥1000 (Referent) | 21/93 | 1.0 | 1.0 | 1.0 | 1.0 |
| 500 - <1000 | 26/112 | 1.0 (0.5 - 1.9) | 0.8 (0.4 - 1.7) | 1.0 (0.5 - 2.0) | 0.8 (0.4 - 1.6) |
| 200 - <500 | 35/116 | 1.3 (0.7 - 2.4) | 1.0 (0.4 - 2.1) | 1.2 (0.7 - 2.4) | 1.0 (0.5 - 2.2) |
| 50 - <200 | 10/43 | 1.1 (0.5 - 2.6) | 0.7 (0.3 - 2.1) | 1.0 (0.4 - 2.4) | 0.8 (0.3 - 2.3) |
| <50 | 1/4 | 1.3 (0.1 - 12.9) | 1.3 (0.1 - 14.1) | 1.4 (0.1 - 14.9) | 1.1 (0.1 - 12.1) |
|  | All subjects 93/368 |  |  |  |  |

^1^Maternal age at delivery, maternal ethnicity and paternal income.

^2^Fuel supply within the 1000 m-buffer, PM_10_, ELF-MF from high-voltage power lines, indoor transformer stations, urban area and arable crop.

**Supplemental Table S4.** Newcastle-Ottawa quality assessment scale (NOS) for included studies: details of score assignment for each included study, divided according to case-control and cohort study design. S-1 through S-4 correspond to the ‘Selection’ questions, C-1 and C-2 correspond to the ‘Comparability’ questions, E-1 through E-3 correspond to the ‘Exposure’ questions reported in Table S2. Letters stand for answers to each question reported in Table S2, and number in parenthesis indicate if the given answer identified a high (1) or low (0) quality rank. Total score is the sum of the score for each answer to the NOS scale. A high score indicates that the study is of high quality.

| **Reference** | **Selection** | | | | **Comparability** | | **Exposure** | | | **Total Score** |
| --- | --- | --- | --- | --- | --- | --- | --- | --- | --- | --- |
|  | *S-1* | *S-2* | *S-3* | *S-4* | *C-1* | *C-2* | *E-1* | *E-2* | *E-3* |  |
| Abdul Rahman 2008 [42] | a (1) | a (1) | b (0) | a (1) | n (0) | n (0) | c (0) | a (1) | a (1) | 5 |
| Brosselin 2009 [44] | a (1) | a (1) | a (1) | a (1) | y (1) | y (1) | a (1) | a (1) | c (0) | 8 |
| Harrison 1999 [46] | a (1) | a (1) | b (0) | a (1) | n (0) | n (0) | a (1) | a (1) | a (1) | 6 |
| Mazzei 2022 [33] | a (1) | a (1) | a (1) | a (1) | y (1) | y (1) | a (1) | a (1) | a (1) | 9 |
| Steffen 2004 [43] | a (1) | a (1) | b (0) | a (1) | y (1) | n (0) | b (1) | a (1) | a (1) | 7 |
| Weng 2009 [45] | a (1) | a (1) | a (1) | a (1) | y (1) | y (1) | b (1) | b (1) | a (1) | 9 |
| This study | a (1) | a (1) | a (1) | a (1) | y (1) | y (1) | a (1) | a (1) | a (1) | 9 |

**Supplemental Figure S1.** Spline regression analysis assessing the risk ratio of childhood leukemia according to distance of child’s residence from petrol station by age at diagnosis (<5 and ≥5 years).


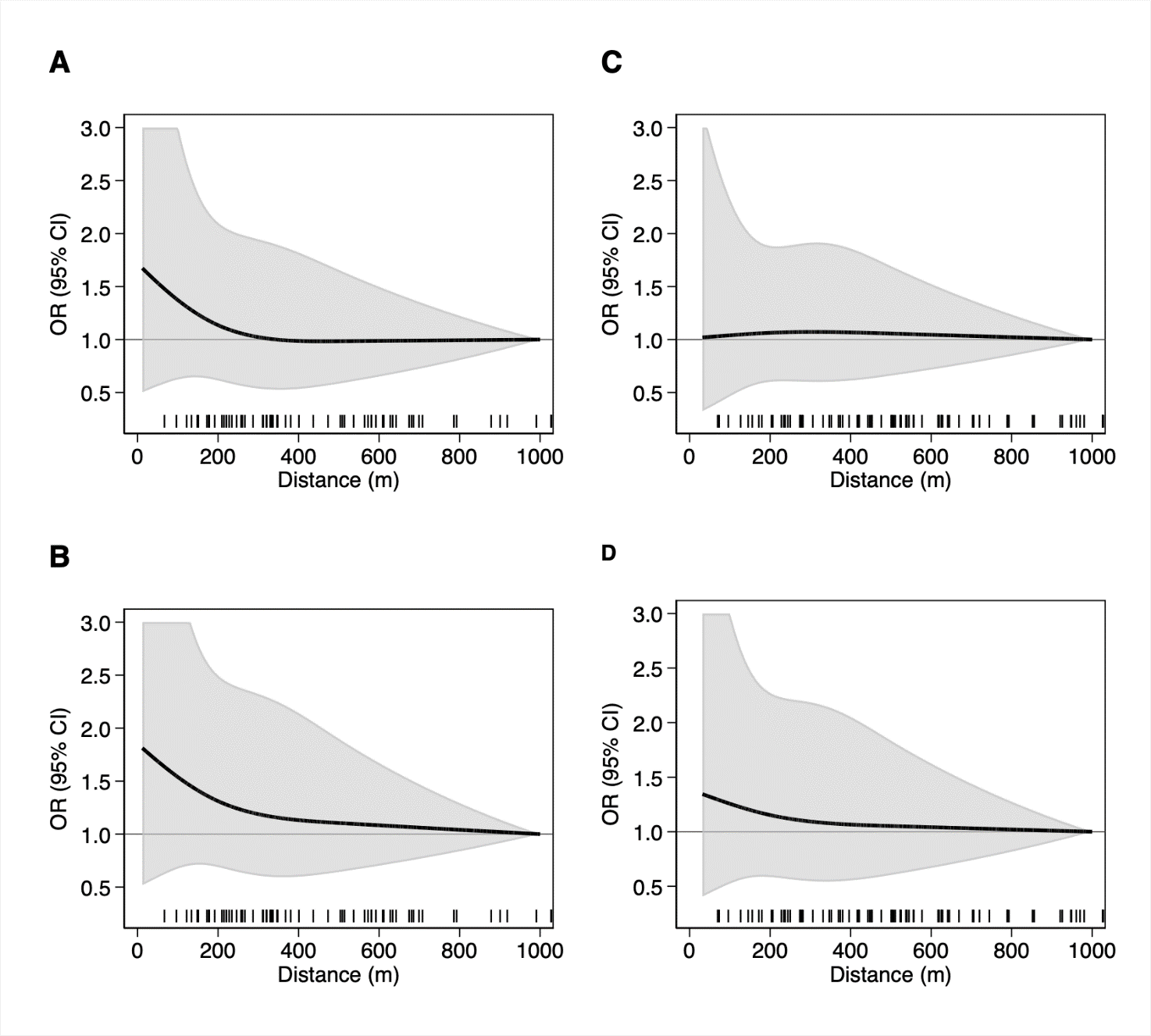


**Supplemental Figure S2**. Flow-chart of included studies.


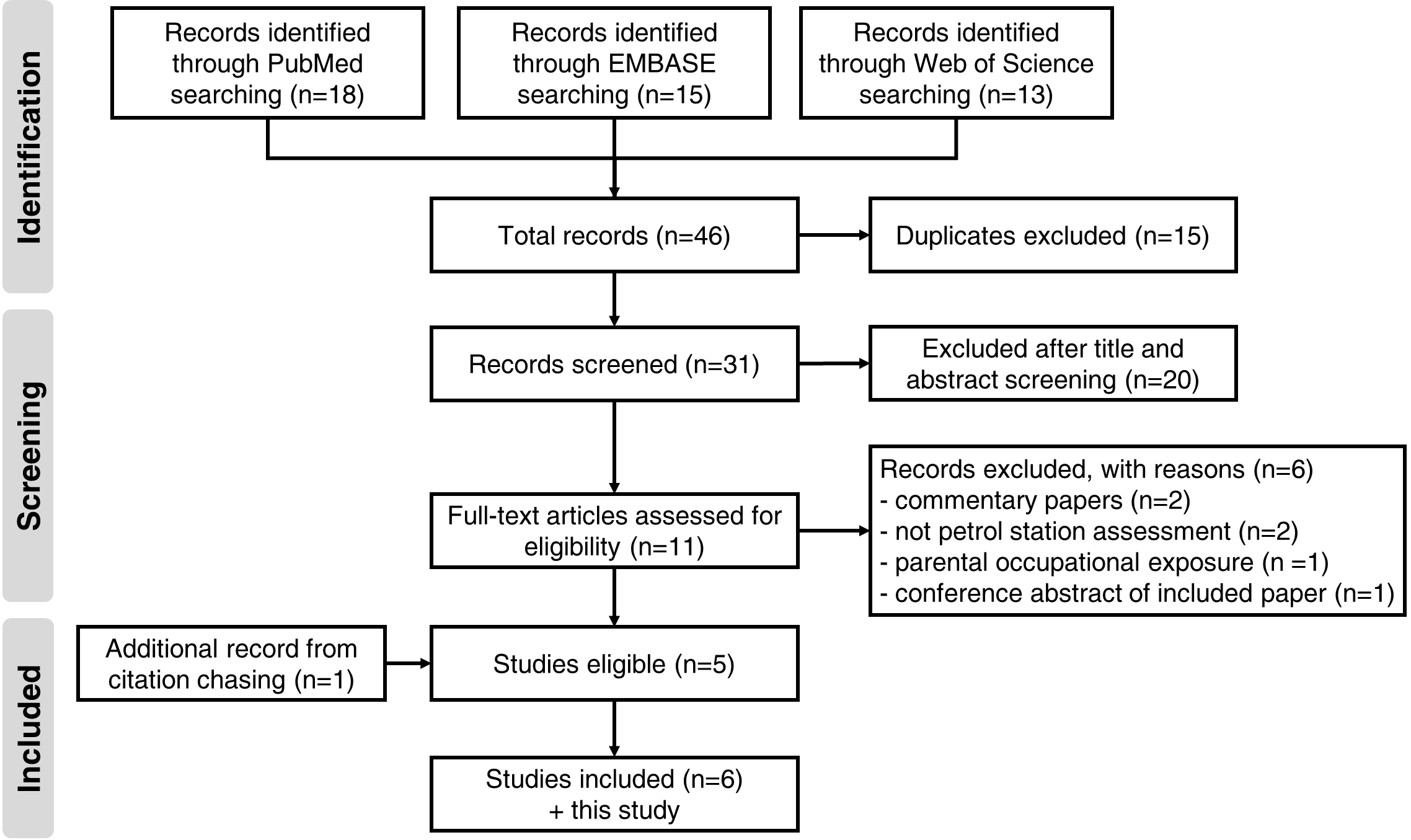


**Supplemental Figure S3.** Forest-plot of the meta-analysis of the association between petrol station exposure and childhood leukemia risk stratified by type of exposure assessment method (i.e. questionnaire and georeferencing data). The area of each grey square is proportional to the inverse of the variance of the estimated log risk ratio (RR) and horizontal lines represent their 95% confidence intervals (CIs). The black diamonds represent the combined RR using the random-effects restricted maximum likelihood (REML) model. The solid vertical line represents RR=1.

**Supplemental Figure S4.** Forest-plot of the meta-analysis of the association between petrol station exposure and childhood leukemia risk after exclusion of one study evaluating any petrol station and repair garage ^41^. The area of each grey square is proportional to the inverse of the variance of the estimated log risk ratio (RR) and horizontal lines represent their 95% confidence intervals (CIs). The black diamond represents the combined RR using the random-effects restricted maximum likelihood (REML) model. The solid vertical line represents RR=1.

**Supplemental Figure S5.** Forest-plot of the meta-analysis of the association between petrol station exposure and childhood leukemia risk restricted to studies with high quality, e.g. Newcastle-Ottawa quality assessment scale (NOS) ≥8. The area of each grey square is proportional to the inverse of the variance of the estimated log risk ratio (RR) and horizontal lines represent their 95% confidence intervals (CIs). The black diamond represents the combined RR using the random-effects restricted maximum likelihood (REML) model. The solid vertical line represents RR=1.

**Supplemental Figure S6.** Funnel plot for publication bias.
